# Detection of Quantitative Trait Loci (QTL) Related to Grilsing and Late Sexual Maturation in Atlantic Salmon (*Salmo salar*)

**DOI:** 10.1007/s10126-013-9530-3

**Published:** 2013-08-04

**Authors:** Alejandro P. Gutierrez, Krzysztof P. Lubieniecki, Steve Fukui, Ruth E. Withler, Bruce Swift, William S. Davidson

**Affiliations:** 1 Department of Molecular Biology and Biochemistry, Simon Fraser University, Burnaby, British Columbia Canada V5A 1S6; 2 Mainstream Canada, 203-919 Island Highway, Campbell River, British Columbia Canada V9W 2C2; 3 Pacific Biological Station, 3190 Hammond Bay Road, Nanaimo, British Columbia Canada V9T 6N7; 4 TRI-GEN Fish Improvement Ltd., 2244 Wilson Road, Agassiz, British Columbia Canada V0M 1A0

**Keywords:** Atlantic salmon, Sexual maturation, Grilsing, QTL, SNP array

## Abstract

In Atlantic salmon aquaculture, early sexual maturation represents a major problem for producers. This is especially true for grilse, which mature after one sea winter before reaching a desirable harvest weight, rather than after two sea winters. Salmon maturing as grilse have a much lower market value than later maturing individuals. For this reason, most companies desire fish that grow fast and mature late. Marker-assisted selection has the potential to improve the efficiency of selection against early maturation and for late sexual maturation; however, studies identifying age of sexual maturation-related genetic markers are lacking for Atlantic salmon. Therefore, we used a 6.5K single-nucleotide polymorphism (SNP) array to genotype five families from the Mainstream Canada broodstock program and search for SNPs associated with early (grilsing) or late sexual maturation. There were 529 SNP loci that were variable across all five families, and this was the set that was used for quantitative trait loci (QTL) analysis. GridQTL identified two chromosomes, Ssa10 and Ssa21, containing QTL related to grilsing. In contrast, only one QTL, on Ssa18, was found linked to late maturation in Atlantic salmon. Our previous work on these five families did not identify genome-wide significant growth-related QTL on Ssa10, Ssa21, or Ssa18. Therefore, taken together, these results suggest that both grilsing and late sexual maturation are controlled independently of one another and also from growth-related traits. The identification of genomic regions associated with grilsing or late sexual maturation provide an opportunity to incorporate this information into selective breeding programs that will enhance Atlantic salmon farming.

## Introduction

The Atlantic salmon (*Salmo salar*) exhibits a great deal of variability in age and size at sexual maturation. This variation is observed both between and within strains and year classes (Garcia de Leaniz et al. 2007; Taranger et al. 2010). The timing of sexual maturation is controlled by a complex process that involves genetic and environmental components (Thorpe and Metcalfe 1998). Internal factors, such as age and lipid reserves, together with external factors, like light abundance, temperature, and food intake, seem to have an impact on the initiation of sexual maturation (Gardner 1976; Herbinger and Friars 1992; Simpson 1992). The interactions of these factors result in the enormous variability in the age of sexual maturation, especially in male Atlantic salmon, which are able to reach maturity at 1 to 7 years of age (Simpson 1992). There is also a genetic component to age at sexual maturation in Atlantic salmon, and estimates of heritabilities (*h* ^2^) for this trait in Atlantic salmon range widely from 0.09–0.17 (Gjerde et al. 1994; Wild et al. 1994; Gjedrem 2000) to 0.39 (Gjerde and Gjedrem 1984) to 0.48 (Gjerde 1984). These observations strongly indicate that age at sexual maturity in Atlantic salmon is a heritable trait. Thus, it should be possible to select for Atlantic salmon that do not become sexually mature as grilse and also for late sexually maturing fish.

In salmon aquaculture, early sexual maturation can be a major economic problem. This is particularly costly in terms of feed and cage space if fish mature as grilse (i.e., after 1 year in a sea cage; Gjedrem 2000). Salmon maturing as grilse have a much lower market value than later maturing individuals (Saunders et al. 1983). The maturation process is energetically expensive, and this is reflected in a decrease in growth rate, lower meat quality, and increased mortality through susceptibility to pathogens (Gjerde 1984; Thorpe 1994). Late-maturing salmon are desired in commercial operations, and to reduce the frequency of grilsing, early maturing Atlantic salmon are discarded as potential breeding candidates (Gjedrem 2012). Selection for the economically important production traits of fast growth and late sexual maturation has been considered problematic as it has been suggested that there is a correlation between the phenotypes of fast growth and early sexual maturation (Thorpe et al. 1983).

Marker-assisted selection has the potential to improve the efficiency of selection for traits such as age of sexual maturation and growth in Atlantic salmon breeding. Genomic regions linked to sexual maturation have been identified in other salmonid species such as rainbow trout and Arctic charr (Easton et al. 2011; Haidle et al. 2008; Küttner et al. 2011; Martyniuk et al. 2003; Moghadam et al. 2007). However, such studies in Atlantic salmon are lacking. The identification of genetic markers independently related to either early or late sexual maturation, especially in male Atlantic salmon, would enable the implementation of selective breeding based on improved genetic selection practices by identifying animals with favorable genotypes. We previously identified QTL for growth at different stages of the production cycle in five families from the Mainstream Canada broodstock (Gutierrez et al. 2012). Here we report QTL for grilsing and late sexual maturation in the same families and show that the genomic locations of these QTL are independent of one another as well as the QTL associated with growth.

## Materials and Methods

### Mapping Families and Phenotype Data

Families were part of a commercial broodstock program developed by Mainstream Canada and based on the Mowi strain of Atlantic salmon, which was derived from a breeding program established using Norwegian populations (Gjedrem et al. 1991). In November/December 2005, 130 single-pair mating families were established. At the fry stage (February 2006), 120 offspring from each family were pooled (15,600 fish in total) and grown communally at the Oceans Farms Hatchery, Vancouver Island. In September/October 2006, 5,000 of the fish were passive integrated transponder (PIT)-tagged, and phenotypic measurements were taken until early 2009. Maturation times in Atlantic salmon were classified as: precocious (≤12 months of age), grilse (36 months of age, at first sea winter (SW)), normally maturing (48 to 60 months of age at second SW or third SW), and late-maturing fish (>60 months) (Gjedrem 2000; Taranger et al. 2010). Sex was recorded during the confirmation of maturation status. The sex of an individual in the late-maturing group could not be ascertained phenotypically; therefore, we predicted the sex of the late-maturing Atlantic salmon using a polymerase chain reaction test, developed by Eisbrenner et al. (2013) that amplifies two exons of the *sdY* gene, which has been shown to be the sex-determining gene in rainbow trout and probably other members of the Salmoninae (Yano et al. 2012, 2013). It should be noted that the precocious Atlantic salmon that matured before 1 year of age were discarded during a standard grading procedure, and so no samples were available from this group.

### DNA Extraction and Parental Assignments

These procedures were as described by Gutierrez et al. (2012). Briefly, DNA was extracted from the adipose fins of the PIT-tagged progeny and the parents used to produce the 130 families (Withler et al. 2005). The DNA was then genotyped using eight microsatellite markers, and parental assignment was carried out as described by Withler et al. (2007).

### SNP Array and Linkage Mapping

The single-nucleotide polymorphism (SNP) data used for this analysis have been described previously (Gutierrez et al. 2012). Five families were selected for SNP genotyping at CIGENE, Norwegian University of Life Sciences, Ås, Norway, using an Atlantic salmon 6.5K Illumina iSelect SNP-array (Kent et al. 2009). Analyses were based on an Atlantic salmon linkage map, which contains ∼5,650 SNP markers and was constructed using genotyping data from 143 families comprising 3,297 fish (Lien et al. 2011). This map contains 29 linkage groups, which were assigned to their specific chromosome number according to the nomenclature established by Phillips et al. (2009). All non-informative markers were removed from the datasets, and subsequently, independent male and female linkage maps were manually created for each family and adjusted to the distances on the SNP map of Lien et al. (2011). Given the differences between the sets of informative markers present in each family, for this study, we restricted the analysis to the 529 markers which are informative in all of the five families. These markers are distributed across all 29 Atlantic salmon chromosomes. We have previously commented on some of the possible reasons why only ∼10% of the markers were variable in all of the five families (Gutierrez et al. 2012). It may reflect the starting population for the Mainstream Canada broodstock or past selection (e.g., primarily for growth in terms of days in salt water to reach 5 kg), or it may reflect the nature of biallelic SNPs. We note that many QTL studies in salmonids have traditionally used microsatellite markers, and these studies tend to have employed fewer variable markers and also fewer families. Even when SNPs were used as the genetic marker, the overall number tested for QTL analysis has been less than 350, and not all of these were variable in each family. Therefore, we consider this study to be robust in terms of what is currently available in the salmonid aquaculture literature.

### QTL Analyses

QTL analyses were performed using a regression-interval mapping method available through the GridQTL portlet (Seaton et al. 2006). GridQTL is a portlet environment (available at http://www.gridqtl.org.uk/) that allows the analysis of extensive datasets. The datasets were analyzed as sib-pairs using the combined genotypic data from the 529 informative markers shared by all families. For the grilsing analysis, given the absence of female grilse, only the genotypic information from males was used. The phenotypic data were scored as a binary trait, i.e., male fish recorded as grilse were scored 0, and male fish recorded as maturing at second or third SW were scored as 1. The same procedure was used for the analysis of late sexual maturation, but both male and female fish were used in this case. Atlantic salmon that did not show evidence of gonadal development at 60 months were considered “late-maturing” and scored as 0. The results were illustrated using the female map as it provides a greater resolution than the male genetic map.

The percentage of phenotypic variance (PEV) explained by the QTL in the sib-pair analysis was calculated as a proportion of the phenotypic variance obtained from the observations. F-statistic values were calculated at 1 cM intervals on each chromosome for each analytical approach to yield the most likely position of the QTL. Empirical chromosome-wide significance thresholds were determined by permutation tests (Churchill and Doerge 1994). The 10,000 permutations were performed at the chromosome-wide level in order to establish the *F*-value thresholds for a *p* < 0.05 and *p* < 0.01. *F*-critical values corresponding to 0.05 > *p* > 0.01 were considered as “suggestive” QTL, whereas those corresponding to *p* < 0.01 were considered “significant” QTL. Those chromosomes which contained QTL that were found to be “significant” were tested for their significance at a genome-wide level of *p* < 0.05 by performing a 1,000 permutation test.

## Results

### QTL Analysis for Male Grilsing

**Fig. 1:**
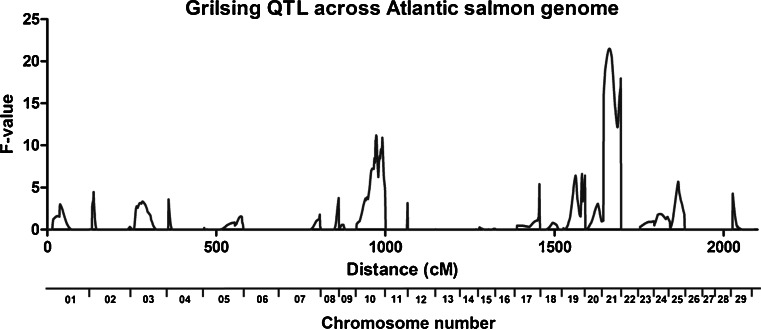
QTL detection for male grilsing across the 29 Atlantic salmon chromosomes

As described previously (Gutierrez et al. 2012), five families were chosen from the 2005 year class of the Mainstream Canada selective breeding program for analysis. Each family contained from five to eight male grilse. No female grilse were observed in any of these families (Table 1). As shown in Fig. 1 and Table 2, QTL analyses restricted to male grilsing revealed the presence of two statistically meaningful QTL, one on chromosome 10 (Ssa10) and the other on chromosome 21 (Ssa21). The QTL on Ssa21 was considered significant (*p* < 0.01), whereas the other on Ssa10 only reached the suggestive level of significance (0.05 > *p* > 0.01). Neither of these QTL reached genome-wide significance, which is in keeping with the relatively small sample size as well as the rather low *h* ^2^ estimates in Atlantic salmon for age at sexual maturation (Gjerde 1984; Gjerde et al. 1994; Wild et al. 1994). The PEV explained by these QTL was 16.7% and 25% in Ssa10 and Ssa21, respectively.

**Table 1:** Number of grilse, normally maturing and late-maturing individuals per family used in this study

Family	# Grilse individuals		# Normally maturing indiv.		# Late maturing indiv.		Total	
	Male	Female	Male	Female	Male	Female	Grilsing analysis	Sexual mat. analysis
F7	5	0	8	25	2	5	13	40
F23	5	0	16	24	3	5	21	48
F76	8	0	9	17	3	3	17	32
F88	5	0	8	20	3	2	13	33
F107	8	0	9	19	6	8	17	42
Total	31	0	50	105	17	23	81	195

**Table 2:** Chromosomes containing male grilsing and late sexual maturation QTL in Atlantic salmon

Trait	Chromosome	Position	*F*-value	LOD	%PEV	RT	AC
Grilsing	Ssa10^a^	60 cM	11.18	2.43	16.7	27 MT	4 MT
Grilsing	Ssa21^b^	17 cM	21.49	4.67	25.0	5 MT	36 BW, K
Late maturation	Ssa18 ^b^	6 cM	20.17	4.38	14.7	–	–

*RT* indicates homologous linkage group in rainbow trout, *AC* indicates homologous linkage group in Arctic charr, *MT* indicates a QTL associated with maturation, *BW* indicates a QTL associated with body weight, *K* indicates a QTL associated with condition factor

^a^Indicates suggestive QTL

^b^Indicates significant QTL

### QTL Analysis of Late Sexual Maturation

**Fig. 2:**
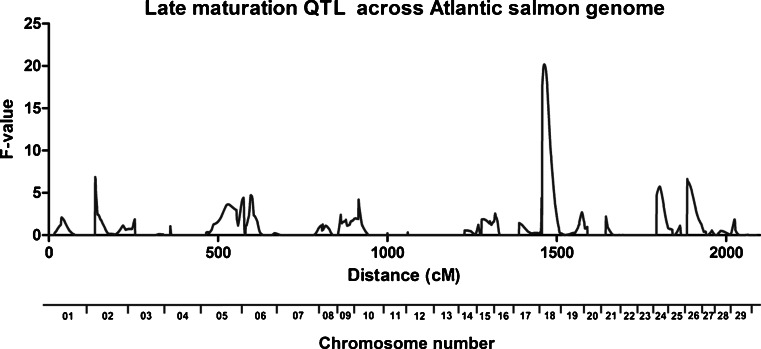
QTL detection for late sexual maturation across the 29 Atlantic salmon chromosomes

From the 195 individuals used in the analysis of late sexual maturation, 40 were fish that did not show gonadal development after their third SW. The sex of the individuals in this latter group was determined based on the presence of two of the four exons of the *sdY* gene (Eisbrenner et al. 2013). Seventeen of the late-maturing 40 fish were male and 23 were female (Table 1). The GridQTL analysis revealed the presence of only one statistically significant (*p* < 0.01) QTL on chromosome 18 (Ssa18), which did not reach genome-wide significance, even though it was the only QTL found for this particular trait (Fig. 2). This QTL explained 14.7 % of the PEV associated with late sexual maturation in these Atlantic salmon families.

## Discussion

Previous studies performed in rainbow trout and Arctic charr have shown an apparent link between QTL for sexual maturation and growth (Haidle et al. 2008; Martyniuk et al. 2003; Moghadam et al. 2007); however, our results indicate a different scenario for Atlantic salmon in the Mainstream Canada breeding program. Here we only detected two QTL associated with male grilsing in Atlantic salmon, on Ssa10 and Ssa21. Previously using the same families, Gutierrez et al. (2012) found genome-wide significant growth-related QTL on chromosomes Ssa02, Ssa07, Ssa13, Ssa09, Ssa17, and Ssa26. Although Gutierrez et al. (2012) found some suggestive evidence for growth-related QTL on Ssa10 and Ssa21, neither reached genome-wide significance, and they mapped to different regions of the chromosomes compared with the grilsing QTL. Moreover, the results of QTL analyses for growth and sexual maturation in the Mainstream Canada broodstock families indicate that sexual maturation is controlled by fewer genomic regions than growth in Atlantic salmon. Therefore, it should be possible to tease apart these traits genetically and so select for each independently using specific genetic markers. A different situation was observed for the QTL for late sexual maturation detected on Ssa18. To date, no growth-related QTL have been reported on this chromosome in Atlantic salmon or on homologous chromosomes in other salmonids (Gutierrez et al. 2012).

Correlations between the phenotypes of growth (as estimated by body weight) and early sexual maturation have been reported in Atlantic salmon (Gjerde 1984; Gjerde et al. 1994; Wild et al. 1994). Moreover, considerable genetic variation in age at sexual maturity has been described in farmed fish species. For example, in Atlantic salmon *h* ^2^ estimates range from 0.09 to 0.17 (Gjerde 1984; Gjerde et al. 1994; Wild et al. 1994) to 0.39 (Gjerde and Gjedrem 1984) to 0.48 (Gjerde 1984) whereas in rainbow trout *h* ^2^ estimates range from 0.12 to 0.35 (Gjerde and Schaeffer 1989; Kause et al. 2003). Nevertheless, selective breeding programs have been effective in increasing body size while also controlling undesired early sexual maturity in farmed fish (Gjedrem 2000), giving clues that these traits are influenced by different genes. Our previous results showed that growth-related QTL were spread across the Atlantic salmon genome (Gutierrez et al. 2012); however, none of the QTL associated with grilsing or late sexual maturation were found in the same chromosomal positions and with the same statistical significance indicating that different genes are associated with these traits.

Comparative genetic mapping in salmonid species has revealed homologous regions of the genomes of Atlantic salmon, rainbow trout, and Arctic charr (Danzmann et al. 2005, 2008; Phillips et al. 2009). Ssa21 corresponds to rainbow trout linkage group 5 (RT-5) (equivalent to chromosome 22) (Phillips et al. 2009) and Arctic charr linkage group 18 (AC-18) (Danzmann et al. 2005), whereas Ssa10 corresponds to RT-8q and RT-27q (equivalent to chromosomes 5q and 2q, respectively) (Phillips et al. 2009) and Arctic charr linkage groups 4, 16, and 40 (AC-4, AC-16, and AC-40) (Danzmann et al. 2005). Genome-wide significant QTL for early male sexual maturation were mapped to RT-8, RT-17, and RT-24, whereas chromosome-wide QTL for this trait was found on RT-3 and RT-19 (Haidle et al. 2008). Martyniuk et al. (2003) did report an association (*p* = 0.043) between microsatellite locus OmyRGT1TUF on RT-5 and precocious maturation and noted that one allele was associated with both a higher body mass and precociousness. However, this association was not considered significant after a sequential Bonferroni correction. Similarly, no *p* values less than 0.01 were identified for microsatellite marker alleles segregating with male sexual maturation in Arctic charr, although SSOSL32 on AC-4 gave a suggestive value of 0.014 (Moghadam et al. 2007).

It is believed that sexual maturation in salmonids depends on the ability of the fish to reach certain developmental thresholds during a “critical period” over the winter/spring (Adams and Thorpe 1989; Thorpe 1994), in a direct relationship with somatic growth and/or energy storage during this period (Taranger et al. 1999). Further investigation of genotype–environment interactions are required to improve Atlantic salmon breeding programs. Photoperiod manipulation using artificial light exposure has proven effective as a means of preventing Atlantic salmon from reaching an anticipated sexual maturation (Endal et al. 2000; Oppedal et al. 2006), and this procedure is used with many other farmed fish (reviewed by Taranger et al. 2010). A clear link between feeding and age of sexual maturation has been described in salmonids (Shearer et al. 2006; Silverstein et al. 1998; Taranger et al. 2010; Thorpe et al. 1990), suggesting that by restricting rations, energy storage and, in particular, lipid stores decrease, thereby delaying sexual maturation (Duston and Saunders 1999; Rowe et al. 1991; Shearer et al. 2006; Shearer and Swanson 2000). This also explains the increasing use of tetradecylthioacetic acid to reduce early sexual maturation in Atlantic salmon (Arge et al. 2012) as it affects the β-oxidation of fatty acids, reducing levels of plasma lipids and adipose lipid stores, and enhancing transport of fatty acids to the liver.

### Candidate Genes

To identify candidate genes associated with the traits of early or late sexual maturation, we made use of the currently available information from the Atlantic salmon genome sequencing project (Davidson et al. 2010), which is publicly available at ASalBase (www.asalbase.org). Most of the SNPs in the 6.5K array correspond to an expressed sequence tag (EST) or are linked to a specific genomic sequence, and these sequences were assigned to a specific whole genome shotgun (WGS) contig by sequence similarity searches. WGS contigs were then annotated using an in-house annotation pipeline (trutta.mbb.sfu.ca) (see Table 3).

**Table 3:** Candidate genes identified in the QTL regions associated with grilsing and late sexual maturation

Trait	SNP_ID	Female map	Male map	WGS contig	BLASTn	Annotation
Grilsing	GCR_cBin22360_Ctg1_176	58.6	2.3	AGKD01085592	*Salmo salar* leukocyte elastase inhibitor (ileu), mRNA	Leukocyte elastase inhibitor (serpin-like) [*Salmo salar*]
Grilsing	GCR_cBin18557_Ctg1_186	58.6	2.3	AGKD01261913	*Osmerus mordax* clone omor-eva-507-083 GDP-L-fucose synthetase putative mRNA, complete cds	PREDICTED: GDP-L-fucose synthase-like [*Oreochromis niloticus*]
Grilsing	GCR_cBin51840_Ctg1_131	59.4	2.3	AGKD01037205	*Salmo salar* clone BHMS423 microsatellite sequence	only ESTs
Grilsing	GCR_cBin11397_Ctg1_74	61.7	2.3	AGKD01171932	No hits	Only ESTs
Late maturation	GCR_cBin5728_Ctg1_254	2.2	1	AGKD01158387	No hits	Only ESTs
Late maturation	GCR_cBin38765_Ctg1_214	8.4	1.3	AGKD01032149	Predicted: *Oreochromis niloticus* myopalladin-like (LOC100700984), mRNA	Predicted: myopalladin-like [*Oreochromis niloticus*]
Grilsing	GCR_cBin17226_Ctg1_170	7.5	1	AGKD01023960	No hits	Predicted: hypothetical protein LOC100002220 [*Danio rerio*] plus ESTs
Grilsing	ESTNV_25113_1234	7.8	1	AGKD01008431	Predicted: *Oreochromis niloticus* formimidoyltransferase-cyclodeaminase-like (LOC100693205), mRNA	Predicted: formimidoyltransferase-cyclodeaminase-like [*Oreochromis niloticus*]
Grilsing	GCR_cBin2393_Ctg1_480	26.2	1.1	AGKD01000933	*Salmo salar* clone ssal-rgf-503-163 E3 ubiquitin-protein ligase UBR3 putative mRNA, partial cds	E3 ubiquitin-protein ligase UBR3 [*Salmo salar*]
Grilsing	GCR_cBin2393_Ctg1_311	26.2	1.1	AGKD01000933	*Salmo salar* clone ssal-rgf-503-163 E3 ubiquitin-protein ligase UBR3 putative mRNA, partial cds	E3 ubiquitin-protein ligase UBR3 [*Salmo salar*]

There are four SNPs linked to the QTL associated with grilsing on Ssa10, of which two could be linked to two genes by a BLASTn search (Altschul et al. 1990) and subsequent sequence annotation. These genes were a leukocyte elastase inhibitor (SERPIN-like) and a GDP-l-fucose synthase-like protein. We cannot speculate how these genes may be related to grilsing in Atlantic salmon. In the case of the SNPs linked to the grilsing QTL on Ssa21, one is associated with a gene encoding a formimidoyl transferase-cyclodeaminase, a protein related to metabolic processes. Two other SNPs (from the same EST) identified E3 ubiquitin-protein ligase (UBR3), a protein that has been shown to have a regulatory role in sensory pathways, including olfaction in mammals (Tasaki et al. 2007). The QTL detected for the late-maturation trait on Ssa18 was linked to two SNPs, but only one of them could be related to a known gene, namely, myopalladin-like gene. Studies in pig and cattle suggest that this gene is associated with meat quality and carcass traits including fat content (Chong et al. 2007; Jiao et al. 2010).

## Conclusions

In this study, we were able to identify a limited number of QTL associated with age at sexual maturation in Atlantic salmon. Moreover, our results suggest that these traits are controlled by genes independently from growth-related traits. Age at sexual maturation is a trait that has been heavily selected and improved after several generations of selective breeding based on family selection and individual selection (Gjedrem 2000; Gjerde 1984; Gjøen and Bentsen 1997). However, these improvements have been based on phenotypic observations, and thus, the identification of genetic markers and genomic regions associated with grilsing and late sexual maturation provides new resources for Atlantic salmon selective breeding programs.
